# High Histological Entropy Is Correlated with Poor Overall Survival and Death Within the First 2 Years in Diffuse Large B-Cell Lymphoma

**DOI:** 10.3390/cancers18142279

**Published:** 2026-07-15

**Authors:** Joaquim Carreras, Yara Yukie Kikuti, Shunsuke Nagase, Giovanna Roncador, Haruka Ikoma, Atsushi Ito, Makoto Orita, Sakura Tomita, Yuki Tanigaki, Akihisa Ueno, Yusuke Kondo, Naoya Nakamura, Yohei Masugi

**Affiliations:** 1 Department of Pathology, School of Medicine, Tokai University, 143 Shimokasuya, Isehara 259-1193, Japan; yy-kikuti@tokai.ac.jp (Y.Y.K.); nagase@tokai.ac.jp (S.N.); ih3822@tokai.ac.jp (H.I.); ito.atsushi.s@tokai.ac.jp (A.I.); orita.makoto.k@tokai.ac.jp (M.O.); sakura.t@tokai.ac.jp (S.T.); tanigaki.yuki.s@tokai.ac.jp (Y.T.); akihisaueno@tokai.ac.jp (A.U.); ky2948@tokai.ac.jp (Y.K.); naoya@tokai.ac.jp (N.N.); masugi@tokai.ac.jp (Y.M.); 2 Monoclonal Antibodies Unit, Spanish National Cancer Research Center (CNIO), Centro Nacional de Investigaciones Oncologicas, C/Melchor Fernandez Almagro, 3, 28029 Madrid, Spain; groncador@cnio.es

**Keywords:** diffuse large B-cell lymphoma, DLBCL, reactive lymphoid tissue, image processing, computer vision, overall survival

## Abstract

**Background/Objectives:** Diffuse large B-cell lymphoma (DLBCL) is an aggressive lymphoma and one of the most common hematological neoplasia. Entropy is a statistical measure of randomness that characterizes the texture of an input image and measures tissue complexity. **Methods:** Image processing and computer vision analysis were performed on a series of 114 diagnostic DLBCL cases and 44 reactive lymphoid tissues stained with hematoxylin and eosin (H&E). Histological entropy was measured to differentiate between reactive lymphoid tissue and DLBCL and predict clinical evolution. At protein level, immunohistochemistry analyzed Ki67, LMO2, MYC, MDM2, CDK6, E2F1, BCL2, CASP8, MYOB, TP53, cPARP, cCASP3, ISY1, TNFAIP8, CSF1R, CD163, PD-L1 and IL-10 markers. Gene expression analysis using the NanoString nCounter PanCancer Immune Profiling Panel was performed in 29 cases. **Results:** In comparison with reactive lymphoid tissue, DLBCL was characterized by lower entropy (7.32 ± 0.16 vs. 6.82 ± 0.44, respectively; *p* < 0.001). Within the DLBCL diagnostic category, higher entropy was associated with poor overall survival and death events within the first 2 years (hazard-risk = 2.4, *p* = 0.004) and lower entropy with a moderate and more favorable outcome (hazard-risk = 0.4, *p* = 0.004). High entropy was also correlated with ECOG performance status ≥ 2, lower protein expression of apoptosis markers of cPARP and cCASP3, and upregulation of specific immuno-oncology genes such as STAT3, BTK, CASP8, CD47, VCAM1, and MYD88. The prognostic value of entropy was independent of the international prognostic index (IPI), Epstein–Barr virus (EBER), and cell of origin (Hans). **Conclusions:** The histological evaluation of entropy is useful for the differential diagnosis of reactive lymphoid tissue and DLBCL and is a predictive factor of DLBCL prognosis.

## Simple Summary

Diffuse large B-cell lymphoma (DLBCL) is an aggressive lymphoma and one of the most common hematological neoplasms. To date, it is not easy to identify DLBCL patients with aggressive clinical evolution and death within the first 2 years after diagnosis, only by using hematoxylin and eosin (H&E) histological images. This study used the entropy function to analyze H&E images of DLBCL to measure randomness, to characterize the texture of an input image, and to measure tissue complexity. Image processing and computer vision analysis were performed on a series of 114 diagnostic DLBCL cases and 44 reactive lymphoid tissues stained with H&E. In comparison with reactive lymphoid tissue, DLBCL was characterized by lower entropy. Within the diagnostic category of DLBCL, higher entropy correlated with poor overall survival and death event within the first two years. In conclusion, the histological evaluation of entropy is useful for both the differential diagnosis of reactive lymphoid tissue and DLBCL and can be used as a predictive factor for DLBCL prognosis.

## 1. Introduction

Diffuse large B-cell lymphoma (DLBCL) is one of the most frequent lymphomas and accounts for around 25 percent of adult non-Hodgkin lymphomas. In the USA, the incidence of DLBCL is around 7 cases per 100,000 persons per year [1,2,3,4].

DLBCL usually presents as symptomatic nodal enlargement in the neck or trunk, but it can originate in extranodal involvement [5]. In one-third of the patients, “B symptoms” are present, including fevers, night sweats, or weight loss [1,2,3,4,6,7,8,9,10,11,12].

DLBCL is a heterogeneous entity with variable clinicopathological characteristics. DLBCL develops from mature B lymphocytes, which morphologically resemble centroblasts or immunoblasts. Based on the cell of origin theory [13], DLBCL can be derived from germinal center B lymphocytes or post-germinal center B cells (also known as activated B lymphocytes), which are characterized by the presence of somatic mutations in the variable region of the immunoglobulin genes (IGVH) [1,2,3,4,6,7,8,9,10,11,12].

The pathogenesis of DLBCL is complex [1,2,3,4,6,7,8,9,10,11,12], including aberrant expression of BCL6, TP53 downregulation, aberrant somatic hypermutation, BCL2 and MYC overexpression, immune evasion, abnormal lymphocyte trafficking, etc. [14]. This multistep process leads to the outgrowth of a malignant B-lymphocyte clone or germinal or post-germinal origin.

The heterogeneity of DLBCL is confirmed by the different identified large B-cell variants, including primary mediastinal large B-cell lymphoma, T-cell-rich large B-cell lymphoma, intravascular B-cell lymphoma, lymphomatoid granulomatosis, Epstein–Barr virus (EBV)-positive DLBCL, DLBCL associated with chronic inflammation, fibrin-associated large B-cell lymphoma, primary large B-cell lymphoma of immune-privileged sites (central nervous system, vitreoretinal, and testis), fluid overload-associated, *IRF4* rearrangement-associated, ALK-positive DLBCL, and double-hit *MYC* and *BCL2* lymphoma (high-grade B-cell lymphoma), among others [6,8].

In two-thirds of the patients, DLBCL is cured with current therapy, particularly if a complete response to first-line therapy is achieved. The prognosis of DLBCL can be predicted using several methods. The International Prognostic Index (IPI) is one of the most used methods and has been validated in patients treated with R-CHOP (rituximab, cyclophosphamide, doxorubicin, vincristine, and prednisone) and other R-CHOP-like combinations. IPI, and variants of R-IPI and NCCN-IPI, include the following variables: age > 60 years, high serum LDH, Eastern Cooperative Oncology Group (ECOG) performance status ≥ 2, clinical stage III or IV, and > extranodal site [15]. The cell of origin status is used to identify activated B-cell (ABC) type cases associated with a worse prognosis. This status can be assessed using gene expression analysis and Hans’ classifier immunohistochemical approach with the evaluation of only three markers, CD10, BCL6, and MUM1 (IRF4) [16]. Other prognostic methods include the assessment of *MYC*, *BCL2*, and *BCL6* translocation status by FISH, deep sequencing, and cell-free plasma DNA analysis.

Achieving a lasting remission for at least 2 years is a good indicator of a favorable long-term prognosis [17,18,19,19,20]. Approximately 30–40% of patients with DLBCL and first-line treatment experience relapsed or refractory disease. In these patients, treatment strategies depend on the timing of relapse (before or above 1 year) [21]. Patients with DLBCL with primary refractoriness or relapse within 1 year are treated with CAR-T (axi-cel or liso-cel) or Glofitamab-GemOx. Patients with relapse after 1 year are treated with immunochemotherapy followed by transplant (high-dose therapy/autologous stem cell transplant) or Glofitamab-GemOx [21].

We have recently described that the survival of DLBCL can be predicted using H&E histological images and deep learning using the 2-year (24 months) cut-off point [22]. This study used a final convolutional neural network based on DarkNet-19 to classify the cases into two prognostic groups and achieved a high performance (test set accuracy of 96.3%). Explainable artificial intelligence (XAI), including Grad-CAM, Image-LIME, and occlusion sensitivity, was used to confirm that the CNN was focusing on the correct areas [22]. However, the “black box” nature of CNNs in medical image classification limits the understanding of their decision-making process [23].

In applied mathematics, the extraction of information from high-dimensional, incomplete, and noisy datasets is generally challenging. The concept of entropy (structural genomic, transcriptomic, network of signal transduction, etc.) has been repeatedly applied to the characteristics of cancer tissue or cells [24,25,26]. In the systems biology narrative, the entropy of signaling and noise is believed to play a major role in the initial oncogenic events [24]. This study focused on the analysis of the texture of the histological images to statistically measure the randomness, in other words, the image entropy. We used grayscale images of DLBCL to measure the entropy and correlated them with the clinicopathological characteristics of the patients. We found that compared to reactive lymphoid tissue, DLBCL was characterized by lower entropy. Within DLBCL cases, DLBCL with a more moderate clinical evolution also had lower entropy values, and aggressive DLBCL had higher entropy.

## 2. Materials and Methods

### 2.1. Patients and Samples

The series included 114 patients with the histological diagnosis of DLBCL and 44 cases of reactive lymphoid tissue. The cases were selected from the tissue bank of the Department of Pathology of Tokai University Hospital. All cases were diagnostic biopsies and de novo DLBCL from 2007 to 2011.

The cases were selected based on the availability of sufficient tissue and size for proper histological diagnosis of DLBCL based on hematoxylin and eosin (H&E) staining, immunophenotype by conventional immunohistochemistry and in situ hybridization, and molecular techniques when required, as described in the current lymphoma classifications [1,2,3]. No specific exclusion criteria were applied, but samples of a minimum size (above 5 mm) were necessary to construct a tissue microarray.

This study was conducted following the guidelines of the Helsinki Declaration for Human Experimentation of the World Medical Association. The study was approved by the Tokai University Institutional Review Board (IRB20/156).

### 2.2. Survival Groups

As previously described in our recent publication of 114 cases [4], two survival groups were created based on the overall survival curve plot and the 2-year inflection point. The aggressive clinical behavior group (*n* = 38; 33.3%) was characterized by a death event before (within) the first 2 years after diagnosis, similar to the concept of progression of disease within 24 months (POD24). The moderate clinical behavior group included the other cases (*n* = 76, 66.7%). The clinicopathological characteristics of the patients were obtained from the Tokai University Hospital intranet card system.

### 2.3. Histological Image Entropy Assessment

Within image processing and computer vision analysis, entropy is a statistical measure of randomness that can be used to characterize the texture of the input image. The analysis was performed using MATLAB R2026a Update 2 (26.1.0.3251617), 5 May 2026.

The dataset included 114 DLBCL and 44 reactive lymphoid tissues. Whole-tissue sections of each case were stained with H&E and digitalized using a NanoZoomer S360 slide scanner (C13220-01, Hamamatsu Photonics K.K., Hamamatsu, Japan). The neoplastic areas were identified and digitally exported into one or several images at 200× magnification and 150 dpi. The images were split into image patches of 224 × 224 × 3 size using PhotoScape v3.7 (website: http://www.photoscape.org/; last accessed on 9 July 2026). All image patches were manually revised to exclude images with less than 80 percent of viable tissue and the absence of diagnostic material based on histopathological criteria: image patches with artifacts such as broken tissue, folded areas, necrosis, incorrectly stained tissue, and smashed or crushed tissue were discarded as previously described [4,5,6,7,8].

All image patches were pooled into three folders: DLBCL Dead within the first 2 years (15,167 image patches), DLBCL Others (27,298 image patches), and reactive lymphoid tissue (326,915 image patches). The three folders were used to perform the path analysis. Later, the patient-based analysis was performed by averaging the entropy values of each patch for each patient separately. $Average=(\sum_{i=1}^{n}Xi)/(n)$. $\sum_{i=1}^{n}Xi\to X1+X2+X3+\ldots +Xn$. n→total number of terms.

H&E image patches were converted from a truecolor image RGB to a grayscale image using the rgb2gray function. This function converts RGB images to grayscale by eliminating the hue and saturation information while retaining the luminance. The RGB2gray function performed the conversion using the GPU. rgb2gray converts RGB values to grayscale values by forming a weighted sum of the R, G, and B components: 0.298936021293775 × R + 0.587043074451121 × G + 0.114020904255103 × B. The coefficients used to calculate grayscale values in rgb2gray are identical to those used to calculate luminance (E’y) in Rec.ITU-R BT.601-7 after rounding to 3 decimal places. Rec.ITU-R BT.601-7 calculates E’y using this formula: 0.299 × R + 0.587 × G + 0.114 × B [9].

The input data were images as grayscale, specified as a numerical array or logical array of any dimension. The entropy function expects images of data types double and single to have values in the range [0, 1]. If an image “I” had values outside the range [0, 1], then the values were rescaled to the expected range using the rescale function. The output was the entropy of image “I”, returned as a numeric scalar [10].

Entropy was defined as -sum(p.*log2(p)) [11], where p contains the normalized histogram counts returned from “imhist”. By default, entropy uses two bins for logical arrays and 256 bins for uint8, uint16, or double arrays. entropy converts any data type other than logical to uint8 for the histogram count calculation, so that the pixel values are discrete and directly correspond to a bin value [12]. Histogram of image data (imhist) calculates the histogram for grayscale image I [13].

The entropy code is shown in Table 1. Raw entropy data is uploaded in the Supplementary File.

### 2.4. Immunohistochemical Procedures

Immunohistochemical stainings and *MYC* and *BCL2* gene rearrangement (translocation) status by FISH were retrieved from our previous publications for some markers [4] and data were reanalyzed. Other markers were newly stained for this study using the Leica Bond Max 2 autostainer following the manufacturer’s instructions (Leica K.K., Tokyo, Japan).

The primary antibodies were the following: Ki67, LMO2, MYC, MDM2, CDK6, E2F1, BCL2, CASP8, MYOB, TP53, cPARP, cCASP3, ISY1, TNFAIP8, CSF1R, CD163, PD-L1, and IL-10. The characteristics of the primary antibodies are shown in Table 2.

### 2.5. Gene Expression Analysis

Gene expression analysis using the NanoString nCounter PanCancer Immune Profiling Panel was available in 29 cases. Whole tissue sections of formalin-fixed paraffin-embedded tissue samples of DLBCL were outsourced to Celgene Corporation (Celgene Corp., Princeton, NJ, USA). After evaluating the histological features to confirm that at least 80% of the sample contained lymphoma cells, RNA extraction was performed using the nCounter platform (NanoString Technologies, Inc., Seattle, WA, USA). This panel included 730 immune-oncology genes and 40 housekeeping genes.

NanoString gene counts calibrated data were normalized to the positive control following the manufacturer’s instructions (NanoString, Seattle, WA, USA). The calibrated data, which is raw data multiplied by the calibration factors, was used for housekeeping genes (hk) gene normalization. The geometric mean of the housekeeping genes for each lane (or each sample) was calculated. The counts for each gene were divided by the lane (or sample) specific geometric mean, and the data were converted to log2 scale. The formula was the following: log2((normData[,i]/hkGeomMeans[i])).

The analysis was the standard, including the calculation of fold change and *p*-values to create heatmap and hierarchical clustering. The hierarchical clustering used the one minus Pearson correlation and average linkage method (software: Morpheus; website location: https://software.broadinstitute.org/morpheus/; last accessed on 8 June 2026).

### 2.6. Statistical Analysis

The analysis included conventional descriptive statistics. Differences between groups included non-parametric tests: independent-samples Kruskal–Wallis Test and pairwise comparisons. Asymptotic significances (2-sided tests) were used. The significance level was 0.050.

In pairwise comparisons, the significance values were adjusted by the Bonferroni correction for multiple tests. Comparison between the two groups was performed using an independent-samples Mann–Whitney U test. Crosstabulation used Chi-square and Fisher’s exact tests. Overall survival was calculated using the Kaplan–Meier and log-rank tests. Survival times ranged from diagnosis to the date of death or last follow-up.

All analyses were performed using IBM SPSS Statistics (version 27.0.1.0, 64-bit edition).

## 3. Results

### 3.1. Clinicopathological Characteristics of the Series

The clinicopathological characteristics of the series are shown in Table 3. In the series of 114 patients, 38 (33.3%) had died within the first 2 years of follow-up (referred to as DLBCL Dead within the first 2 years). The Others group corresponded to 76 patients (66.7%). This DLBCL Others group experienced a death event after 2 years in 16 patients (29.6%). DLBCL Dead within the first 2 years had a mean survival time of 7.6 months vs. 140 months in the DLBCL Others group (Kaplan–Meier and log-rank test (Mantel–Cox), *p* < 0.001).

DLBCL Dead within the first 2 years were characterized by several clinicopathological variables usually associated with poor prognosis of DLBCL, including high LDH, ECOP performance status ≥ 2, international prognostic index (IPI) high or high–intermediate, low clinical response to treatment (RCHOP or RCHOP-like), higher death events, non-germinal center B-cell-like by the Hans algorithm, positivity by Epstein–Barr Virus (EBV), and an immune microenvironment characterized by high infiltration of CD163-positive tumor-associated macrophages (TAMs) (all *p*-values < 0.05).

### 3.2. Assessment of Histological Entropy in Schematic Images

The function of entropy uses a grayscale image to return the entropy value, which is a statistical measure of randomness that can be used to characterize the texture of the input image. Entropy is a method to analyze the complexity of an image.

**Figure 1:**
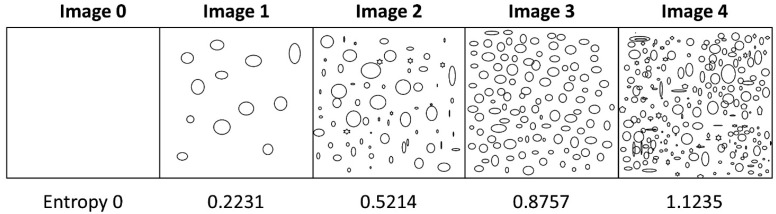
Entropy measurement in the schematic set. In image processing and computer vision, entropy is a function that measures the randomness of a grayscale image. Entropy is a statistical measure of randomness that can be used to characterize the texture of the input measure. This study used entropy to measure the complexity of the reactive lymphoid tissue and DLBCL, but first, the analysis was tested in five different images, 0–4, with increased complexity (randomness).

**Figure 2:**
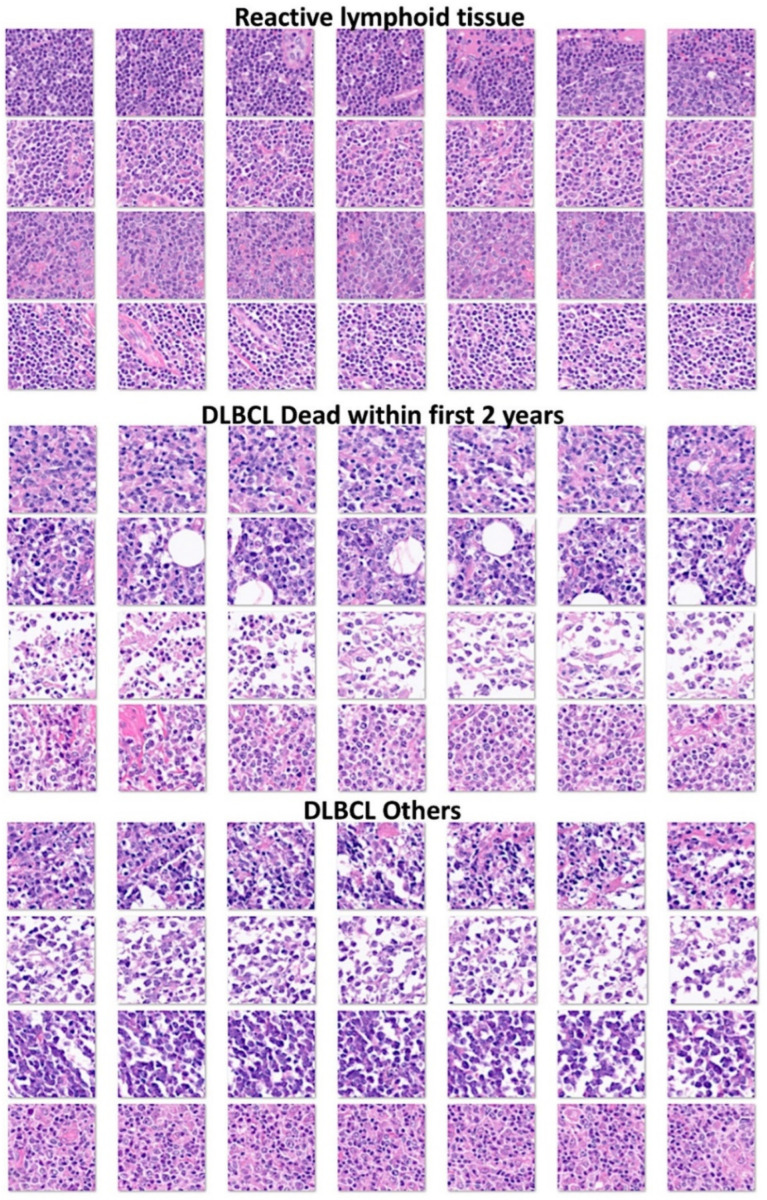
Hematoxylin and eosin images. This figure shows examples of image-patches of reactive lymphoid tissue, DLBCL Dead within the first 2 years, and DLBCL Others. Although it is possible to diagnose and differentiate between reactive lymphoid tissue and DLBCL by a histopathologist, it is difficult to differentiate between the 2 DLBCL groups using a conventional optical microscope. Therefore, entropy analysis was performed to analyze the tissue structure and complexity (randomness).

To validate this assumption and methodology, entropy was assessed in four different test images (Figure 1 and Figure 2). The experiment aimed to show if entropy increased with image complexity (randomness). For each image, the measurement was repeated 10 times, and we confirmed that the value was the same in each measurement. The results were the following: image 0, 0 entropy; image 2, 0.5214; image 3, 0.8757; and image 4, 1.1235. Therefore, we confirmed that entropy increased with image complexity (randomness).

### 3.3. Assessment of Histological Entropy in Reactive Lymphoid Tissue and DLBCL

Next, entropy was measured in all the image patches of reactive lymphoid tissue and DLBCL. The number of image patches was the following: DLBCL (*n* = 42,465) and reactive lymphoid tissue (*n* = 326,915) (total *n* = 369,380). Within the DLBCL category, the frequencies were DLBCL Dead within the first 2 years (*n* = 15,167) and DLBCL Others (*n* = 27,298). For statistical purposes, the entropy values of the image patches were averaged for each case (i.e., patient-based analysis).

**Figure 3:**
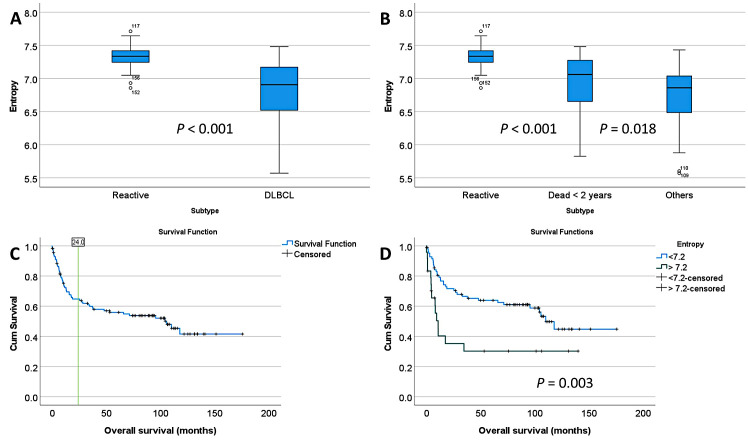
Entropy measurement between groups. DLBCL is a neoplasm of medium or large-sized B lymphocytes with a diffuse growth pattern. Lymph nodes demonstrate partial or, more commonly, total architectural effacement by diffuse proliferation of lymphoid cells. (**A**) In comparison to reactive lymphoid tissue, DLBCL was characterized by lower entropy (randomness) (Case-based analysis). (**B**) In comparison to DLBCL Dead within the first 2 years, DLBCL Others had lower entropy measurement (*p* = 0.018). Reactive lymphoid tissue (7.32 ± 0.16), DLBCL Dead within the first 2 years (6.94 ± 0.44), and DLBCL Others (6.75 ± 0.0.42) (all comparisons were statistically significant, *p* < 0.001). (**C**) The overall survival curve of this series of DLBCL had a point of inflection at 2 years (24 months). (**D**) DLBCL cases with high entropy were associated with poor overall survival (*p* = 0.003). Of note, high entropy correlated with death within the first 2 years (*p* < 0.05) using crosstabulations.

In comparison to reactive lymphoid tissue, DLBCL was characterized by lower entropy: 6.82 ± 0.44 vs. 7.32 ± 0.16 (Independent-sample Mann–Whitney U test, *p* < 0.001) (Figure 3A).

In comparison to DLBCL Others, DLBCL Dead within the first 2 years were characterized by higher entropy: 6.94 ± 0.44 vs. 6.75 ± 0.42 (Independent-sample Mann–Whitney U test, *p* = 0.015 (Figure 3B).

The correlation between entropy and DLBCL overall survival was analyzed, and a cutpoint was searched. Two cutpoints with 33.33% of the interval width were set at ≤6.87, 6.88–7.25, and >7.26, and the final optimal cutpoint was set at 7.2. The frequencies were as follows: <7.2, *n* = 85 cases (78%), and >7.2, *n* = 24 (22%). Univariate overall survival analysis using the Kaplan–Meier and Log–rank test showed that cases with high entropy (>7.2) were associated with poorer survival (*p* = 0.003) (Figure 3C,D). Univariate COX regression analysis confirmed that high entropy (>7.2) was associated with a hazard risk of 2.4 (95% confidence interval 1.3–4.4, *p* = 0.004).

Entropy levels correlated with the DLBCL groups, and the data are shown in Table 4. Entropy > 7.2 correlated with DLBCL Dead within the first two years, and entropy < 7.2 to DLBCL Others (*p* = 0.002).

**Figure 4:**
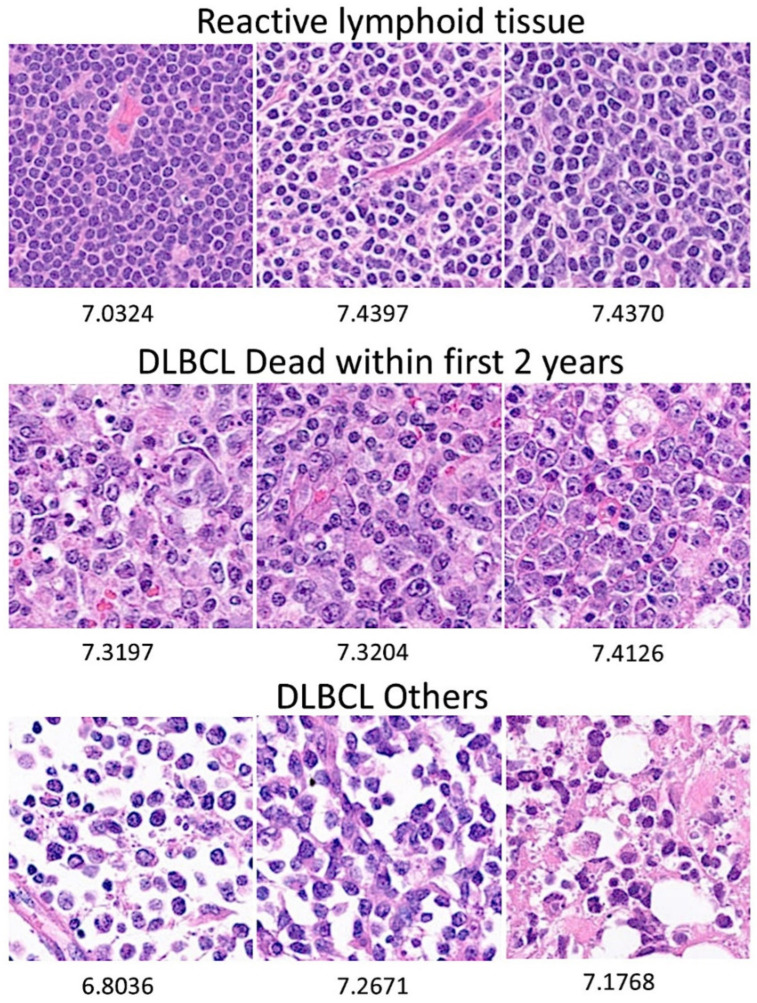
Images and entropy measurement between groups. Overall, DLBCL Dead within the first 2 years was characterized by higher entropy than the DLBCL Others group.

**Figure 5:**
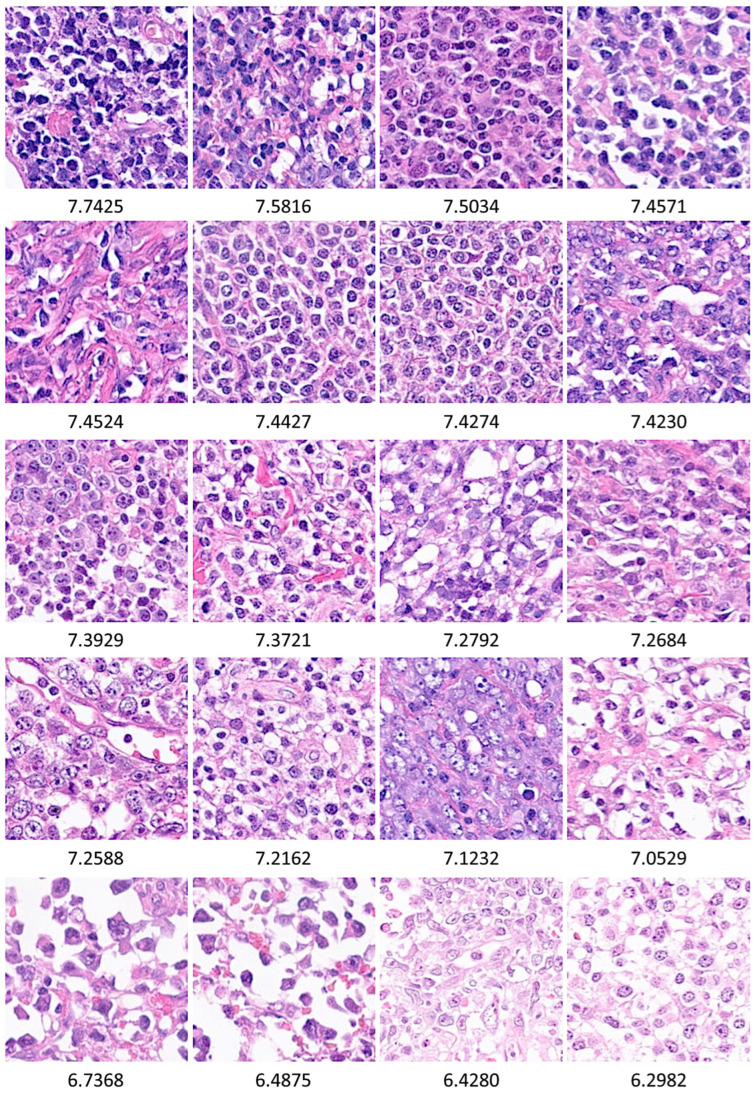
Images and entropy measurement in the DLBCL Dead within the first 2 years group. In comparison with DLBCL Others, the DLBCL Dead within the first 2 years group was characterized by higher entropy (*p* = 0.01) (patient-based analysis). DLBCL cases with high entropy were associated with poor overall survival (*p* = 0.003) (patient-based analysis). The entropy value is shown below each image.

**Figure 6:**
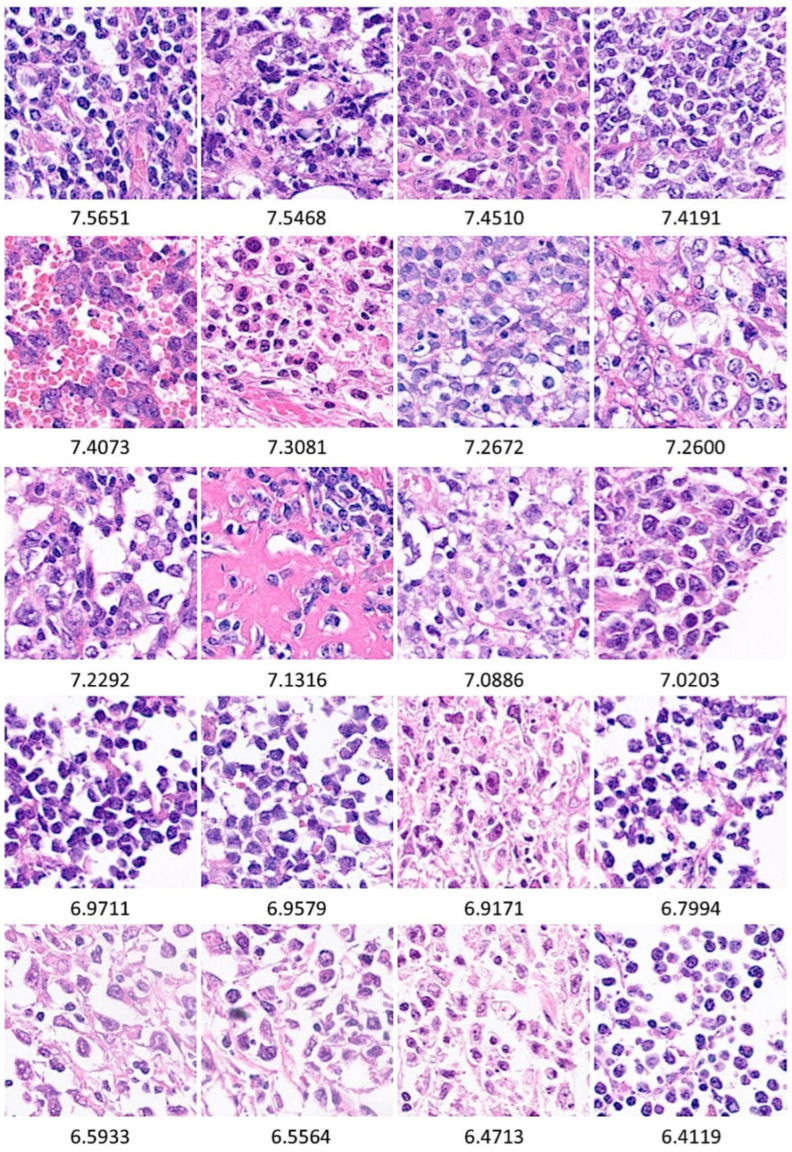
Images and entropy measurement in the DLBCL Others group. In comparison with DLBCL Dead within the first 2 years, DLBCL Others was characterized by lower entropy (*p* = 0.01) (patient-based analysis). DLBCL cases with low entropy were associated with better (moderate) overall survival (*p* = 0.003) (patient-based analysis). The entropy value is shown below each image.

Examples of entropy levels in specific image patches of reactive tissue and DLBCL are shown in Figure 4, Figure 5 and Figure 6.

**Figure 7:**
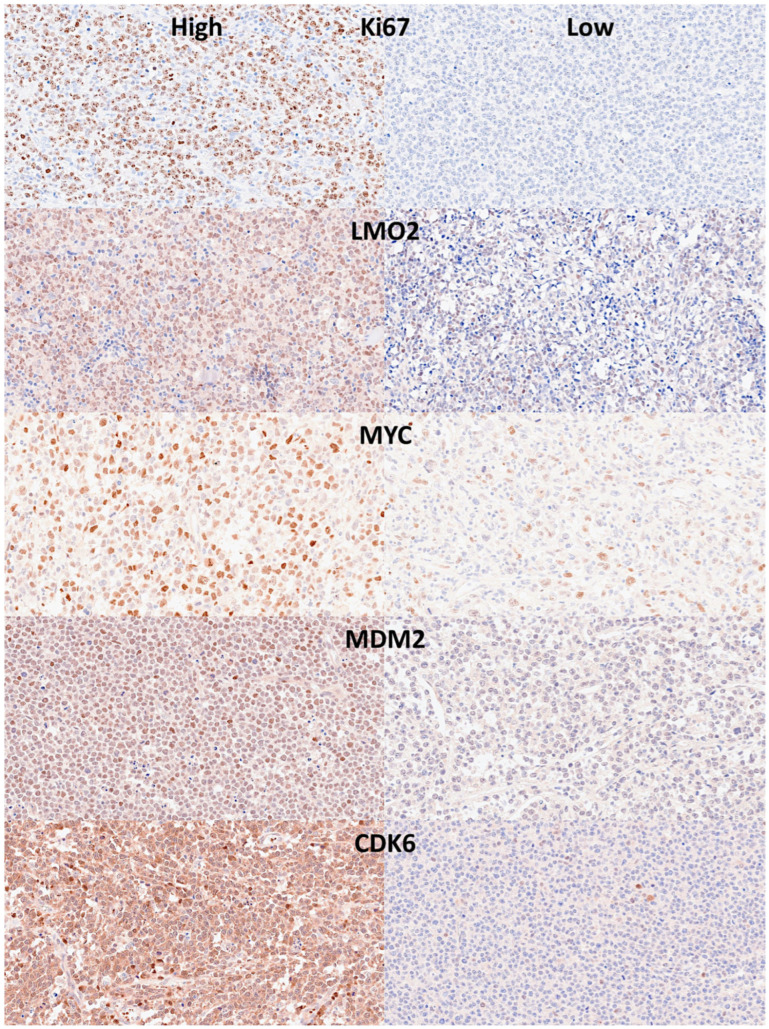
Immunohistochemical analysis 1. Further analysis included the correlation of entropy levels and several clinicopathological markers. Cases with high entropy correlated with Dead within the first 2 years, ECOG performance status ≥ 2, and lower apoptotic markers of cPARP and cCASP3 evaluated by immunohistochemistry. Original magnification 400×.

**Figure 8:**
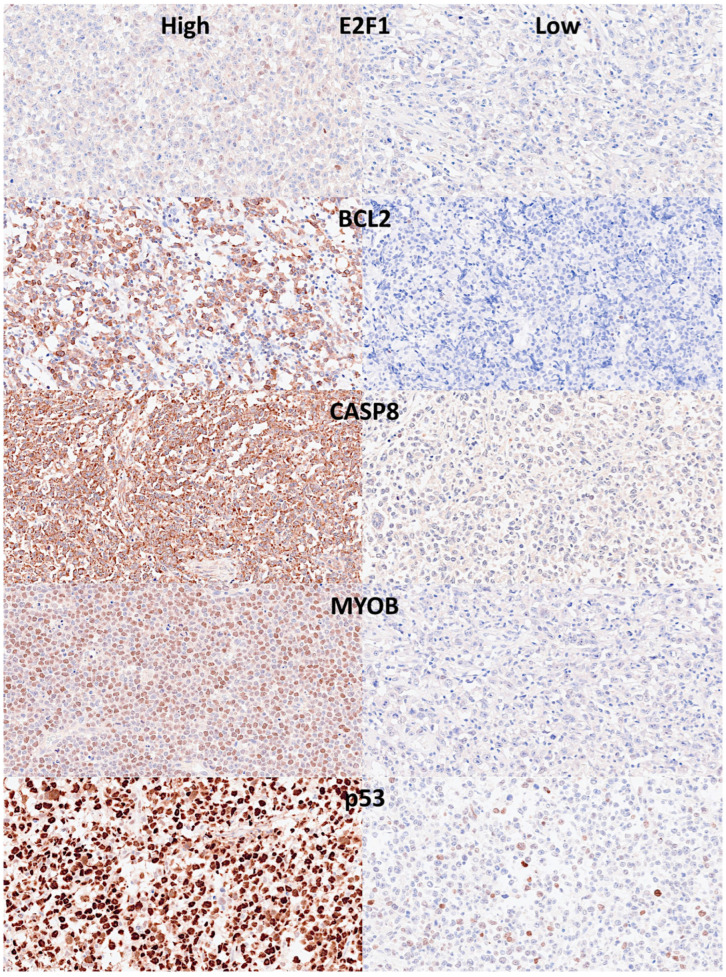
Immunohistochemical analysis 2. Immunohistochemical analysis. Further analysis included the correlation of entropy levels and several clinicopathological markers. Cases with high entropy correlated with death within the first 2 years, ECOG performance status ≥ 2, and lower apoptotic markers of cPARP and cCASP3 evaluated by immunohistochemistry. Original magnification 400×.

**Figure 9:**
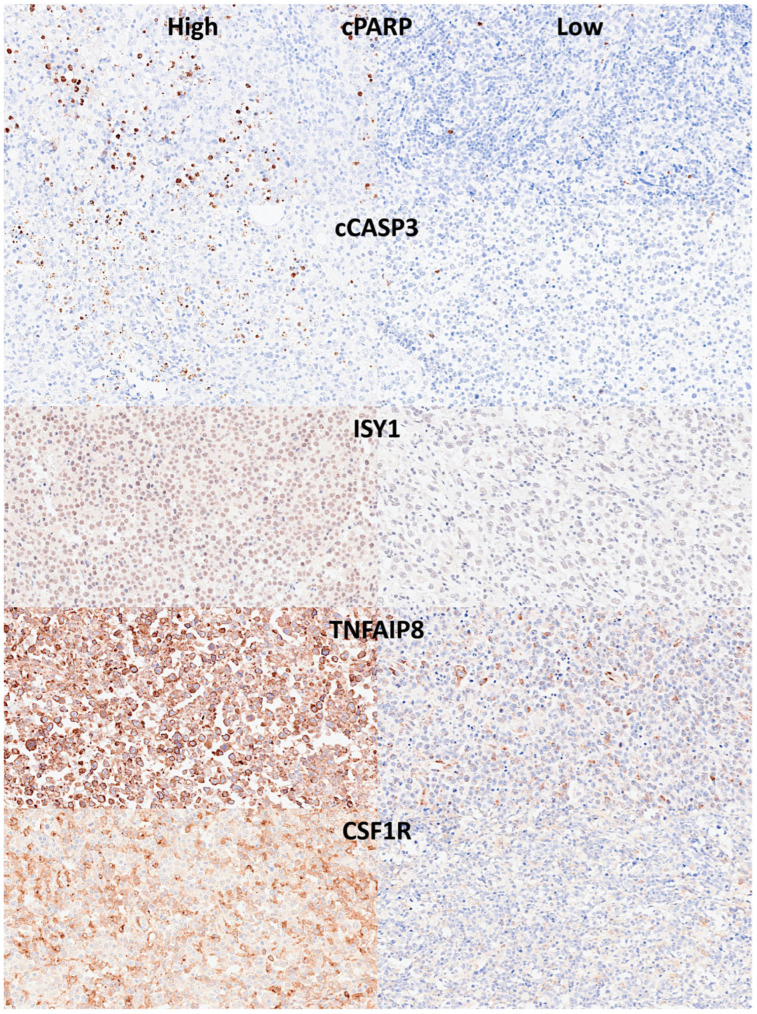
Immunohistochemical analysis 3. Immunohistochemical analysis. Further analysis included the correlation of entropy levels and several clinicopathological markers. Cases with high entropy correlated with death within the first 2 years, ECOG performance status ≥ 2, and lower apoptotic markers of cPARP and cCASP3 evaluated by immunohistochemistry. Original magnification 400×.

**Figure 10:**
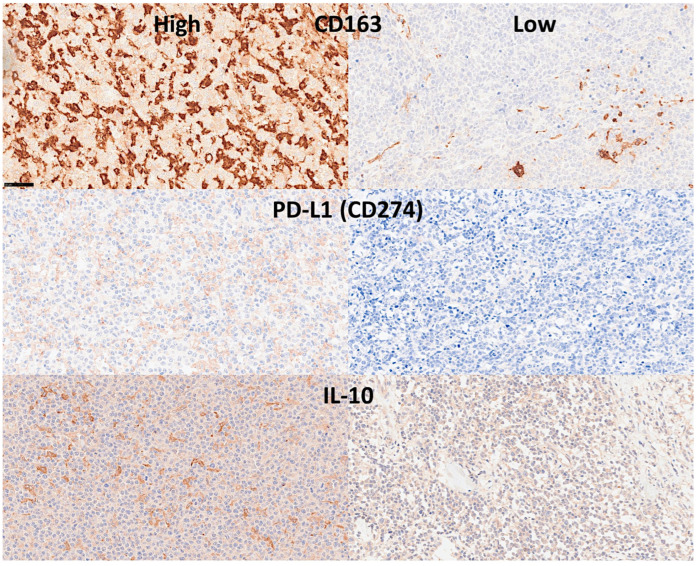
Immunohistochemical analysis 4. Further analysis included the correlation of entropy levels and several clinicopathological markers. Cases with high entropy correlated with death within the first 2 years, ECOG performance status ≥ 2, and lower apoptotic markers of cPARP and cCASP3 evaluated by immunohistochemistry. Original magnification 400×. Scale bar = 50 um.

Further analysis included the correlation of entropy levels with several clinicopathological characteristics and several pathological markers. Cases with high entropy correlated with death within the first 2 years, ECOG performance status ≥ 2, and lower apoptotic markers of cPARP and cCASP3 evaluated by immunohistochemistry (Table 5 and Table 6, and Figure 7, Figure 8, Figure 9 and Figure 10).

Table 7 shows the Cox regression multivariate analysis for overall survival: entropy, Epstein–Barr virus (EBER), and cell of origin (Hans) kept the prognostic value when included in the same multivariate analysis.

### 3.4. Differential Gene Expression Between High and Low Entropy DLBCL Groups

**Figure 11:**
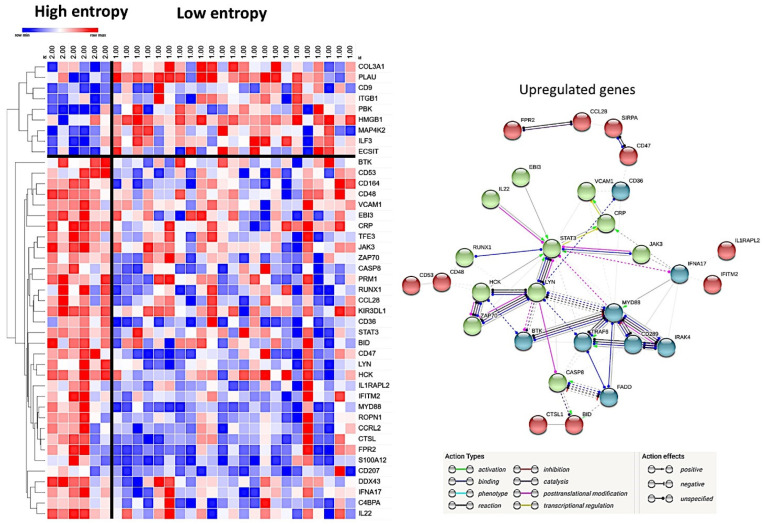
Gene expression analysis. Gene expression analysis was available in 29 cases, including 6 cases with high entropy and 23 cases with low entropy. The gene expression analysis used an immune profiling (pan-cancer immuno-oncology) panel. Among the 730 genes of the panel, statistical differences were found in 43 genes: 34 were upregulated and 9 downregulated in the high entropy group. A functional network association analysis highlighted the “hub genes” that are potentially the most relevant among the upregulated genes.

Gene expression analysis using the immune profiling (pan-cancer immuno-oncology panel) was performed in 6 cases of high entropy and 23 cases of low entropy. Among the 730 genes of the panel, statistical differences were found in 43 genes: 34 were upregulated and 9 downregulated in the high entropy group. Among the upregulated genes, several relevant genes were found, such as *STAT3* (chemokines, regulation), *BTK* (adaptive and immune response), *CASP8* (apoptosis, innate immune response), *CD47* (macrophage functions, regulation), *VCAM1* (adhesion, regulation of immune response), *MYD88* (innate immune response, toll-like receptor), *ZAP70* (adaptive immune response), and *CRP* (acute-phase response, phagocytosis), have been previously identified in lymphoid neoplasia and other inflammatory conditions. The heatmap and functional network association analysis of the upregulated genes are shown in Figure 11.

## 4. Discussion

This study focused on the analysis of histological features of DLBCL and reactive lymphoid tissue using entropy, which is an image processing and computing vision analysis function. Entropy is a statistical measure of randomness that can be used to characterize the texture of an image. It can also be used to analyze the histological complexity of a tissue.

A major question when making a diagnosis of a lymph node biopsy is whether the process is benign or malignant [5,63]. Distinction between reactive and malignant lymphoid proliferation is based on several variables, including abnormal architecture (effacement of architecture), evidence of invasion (invasion of epithelium/gland, interstitial tissues, blood vessel walls, atypical lymphoid cells in interfollicular areas, destruction of mantle zones, or colonization of lymphoid follicles), and cytological atypia (large cells, irregular nuclei, granular chromatin, clear cells, and aberrant immunophenotype) [64].

This study included 44 cases of reactive lymphoid tissue, including both tonsils and lymph nodes. According to the architectural histologic pattern, there are four types of reactive lymphadenopathies: follicular/nodular, sinus, interfollicular or mixed, and diffuse. However, several compartments may be involved in a single case [5,63].

The classification of reactive lymphadenopathies includes four categories [5,63]: follicular hyperplasia and autoimmune disorders, characterized by a follicular and nodular pattern; sinus histiocytosis, such as Whipple disease [65]; interfollicular or mixed pattern, such as Kimura disease [66], Kikuchi disease [67], and Lupus; and diffuse pattern, including cytomegalovirus and infectious mononucleosis. Follicular hyperplasia is the most frequently made diagnosis and is characterized by multiple large, irregular follicles with germinal centers. Other characteristics include the presence of polarization, a starry-sky pattern with multiple tangible body macrophages, evidence of mantle zones, the presence of plasma cells within the follicles, the lack of BCL2 expression evaluated by immunohistochemistry in the B lymphocytes of follicles, and the absence of translocation (14;18)(q32;q21) by FISH [68], PCR, or next-generation sequencing techniques [69,70,71]. Overall, reactive lymphoid tissue is characterized by being heterogeneous.

Conversely, DLBCL has specific histological characteristics. DLBCL demonstrates partial or, more commonly, a total architectural effacement of the lymph node by diffuse proliferation of medium to large lymphoid cells [3,63,72,73,74]. The neoplastic B lymphocytes fit into three common morphological variants, namely centroblastic, immunoblastic, and anaplastic variants. Centroblastic morphology is the most common. Centroblasts are medium-sized to large lymphoid cells with round and vesicular nuclei with fine chromatin [1]. The immunoblastic variant has >90% immunoblasts characterized by a single centrally located nucleolus and basophilic cytoplasm. Anaplastic variant includes large cells with bizarre pleomorphic nuclei that may resemble Hodgkin/Reed–Sternberg cells [75]. In some cases, the anaplastic variant may resemble undifferentiated carcinoma [76,77,78,79,80]. Other rare morphological variants include myxoid stroma, fibrillary matrix, and spindle-shaped cells [81,82,83], multilobated [84,85,86], and signet ring cells [87,88].

All these histological characteristics can potentially be analyzed using image processing and computer vision strategies. We recently successfully performed image classification of benign and neoplastic conditions using convolutional neural networks and deep learning [4,5,6,7,8]. However, without a complete understanding of how deep learning models make decisions and a lack of control over the internal decision-making processes, it is difficult to implement artificial intelligence (AI) methods in critical applications and environments [89]. As neural network models are described as a “black box”, several techniques have been developed to explain how they work. These techniques are known as eXplainable Artificial Intelligence (XAI). In our studies, we have successfully used Grad-CAM, image LIME, and occlusion sensitivity [4,5,6,7,8], but a complete understanding of how deep learning models work and make decisions is elusive. In this study, we used a different approach and focused on computer vision analysis of entropy, which was previously applied in the study of acute lymphoblastic leukemia classification [90]. We found that it was feasible to differentiate between reactive lymphoid tissue and DLBCL. Entropy level (randomness) was lower in DLBCL, which could represent a measurement of monoclonality of the neoplastic B lymphocytes and diffuse effacement of the lymphoid tissue architecture.

DLBCL is an aggressive B-cell malignancy and one of the most common lymphomas. Under current treatments, two-thirds of patients are expected to have long-term survival. However, in one-third of patients, the disease behaves aggressively [1,2,3,73]. First-line treatment of DLBCL includes R-CHOP in the case of the GCB subtype, Pola-R-CHP in the ABC subtype, and DA-R-EPOCH in double-hit *MYC* and *BCL2* [76]. Second-line treatment differs depending on the time of progression from diagnosis. Early progression (<1 year from diagnosis) can be treated with CAR T-cell therapy (including bridging therapy). Late progression (>1 year from diagnosis) includes salvage therapy and autologous stem cell transplant [76]. If unsuccessful, third-line therapy will be applied, including CAR T, bispecific antibodies, antibody-drug conjugate combinations, monoclonal antibody combinations, novel agents/clinical trials, and supportive/palliative care [91,92,93].

This study divided the DLBCL patients into two groups according to the overall survival: DLBCL patients who experienced death events within the first 2 years after diagnosis and the others. This division is compatible with the early and late progression algorithms [76,91,92,93]. The correlation with clinicopathological characteristics showed that patients with DLBCL with the lowest entropy were associated with a moderate overall survival, but patients with DLBCL with higher entropy were associated with aggressive clinical evolution and death within the first 2 years. Therefore, the evaluation of histological entropy may be useful in the assessment of risk in the future.

Finally, the gene expression analysis allowed us to identify which genes and immuno-oncology pathways were relevant in this model and to know which genes were upregulated and downregulated in the cases with high entropy. Relevant genes were *STAT3*, *LYN*, and *MYD88*, which are genes known to be relevant in DLBCL pathogenesis [94,95,96].

## 5. Limitations

A limitation of this study is the relatively small series of cases and the lack of an external validation set. In the future, a larger series of DLBCL cases could be analyzed.

## 6. Conclusions

In comparison to reactive lymphoid tissue, DLBCL is characterized by lower histological entropy (randomness). Within the DLBCL diagnostic category, higher entropy is associated with aggressive clinical evolution and death within the first 2 years, and lower entropy is associated with a moderate and more favorable outcome.

## Figures and Tables

**Table 1:** Entropy code.

**% Basic code** I = imread(“***”) A = rgb2gray(I); J = entropy(A)	**% The code was the following** imageLoc = “***”; ds = imageDatastore(imageLoc); for i = 1:length(ds.Files) I = readimage(ds, i); A = rgb2gray(I); J(i) = entropy(A); end **% Display the entropy values for each image** disp(J);	**% Plot the entropy values** figure; bar(J); xlabel(‘Image Index’); ylabel(‘Entropy’); title(‘Entropy of Images in ***’);

**Table 2:** List of primary antibodies.

Marker	Target	Clone, Company
Ki67	Cell proliferation and growth [14,15,16]	RTU, MM1, Leica
LMO2	Nuclear marker in normal germinal-center (GC) B cells and GC-derived B-cell lymphomas [17,18]	299B, created by the Monoclonal Antibodies Unit, Centro Nacional de Investigaciones Oncologicas, CNIO, Madrid, Spain
MYC	Proto-oncogene, transcription factor with a wide array of functions, including cell cycle, apoptosis, DNA damage response, and hematopoiesis [19,19]	Y69, Abcam
MDM2	An important regulator of the tumor suppressor p53 [20,21,22]	IF2, Invitrogen
CDK6	Cyclin-dependent kinase-6 is important in the progression of cells from the G1-phase to the S-phase of the cell cycle [23,24]	98D, CNIO
E2F1	A transcription factor with a role in cell cycle progression from G1 to S phase. Upregulated in many types of neoplasia [25]	Agro368V, CNIO
BCL2	Human proto-oncogene. Suppresses apoptosis and regulates cell death [26,27,28,29]	bcl2/100/D5, Leica
CASP8	Apoptosis, initiator caspase of extrinsic apoptosis [30,31,32]	11B6, NCL-CASP-8, Novocastra (Leica)
MYOB	A-myb/B-myb/C-myb. Nuclear proteins that function as transcriptional transactivators. B-cells at the germinal centers express A-myb. C-myb is predominantly expressed in immature hematopoietic cells [33,34,35,36]	DANI51, CNIO
TP53	Tumor protein 53. Tumor suppressor transcription factor that controls cell cycle, apoptosis, senescence, and DNA repair. Mutated in many human cancers [37]	D0-7, Novocastra, Leica
cPARP	Cleaved PARP (Asp214). Large fragment (89 kDa) of human PARP1 produced by caspase cleavage. Apoptosis [38,39].	Asp214, D64E10, Cell Signaling Technology (CST)
cCASP3	Caspase-3 is a protease with a major role in the execution of apoptosis. Cleaved caspase-3 (Asp175) detects a large fragment of activated caspase-3. Responsible for the proteolytic cleavage of many key proteins, such as PARP1 [40].	Asp175, #9661, CST
ISY1	Spliceosome-associated RNA-binding protein that functions in pre-mRNA splicing and in the selective biogenesis of microRNAs [41,42]	#NBP1-81864, Novus Biologicals
TNFAIP8	Regulation of apoptosis, both positive and negative regulation, is an immuno-oncology marker [30,31,43,44,45,46]	#14559-MM01, Sino Biological
CSF1R	M2-like TAMs [47,48,49,50]	FER216D, CNIO
CD163	M2-like TAMs [51,52,53,54]	10D6, Novocastra, Leica
PD-L1 (CD274)	Immuno-oncology [55,56,57,58]	E1J2, CST
IL-10	Immune-regulatory, M2c-like TAMs [55,59,60,61,62]	LS-B7432, Lifespan Bioscience

CNIO, Centro Nacional de Investigaciones Oncológicas (Spanish National Cancer Research Center). Leica, Novocastra antibodies, Leica Biosystems K.K., Tokyo, Japan. Abcam, Abcam K.K., Tokyo, Japan. Invitrogen, Thermo Fisher Scientific, Invitrogen Japan K.K., Tokyo, Japan. CST, Cell Signaling Technology K.K., Tokyo, Japan. Novus Biologicals LLC, Centennial, CO, USA. Sino Biological Inc., Beijing, China. Lifespan Bioscience, Seattle, WA, USA.

**Table 3:** Clinicopathological characteristics.

	Total	Dead Within the First 2 Years	Others	*p*-Value
Frequency	114 (100%)	38/114 (33.3%)	76/114 (66.7%)	N/A
Entropy	6.80 ± 0.61	6.83 ± 0.63	6.78 ± 0.59	<0.001
Clinical features				
Age > 60 years	81/114 (71.1%)	30/81 (37.0%)	51/81 (63.0%)	0.273
Sex male	60/114 (52.6%)	19/60 (31.7%)	41/60 (68.3%)	0.697
Location				
Nodal (+spleen)	58/114 (50.9%)	16/58 (27.6%)	42/58 (72.4%)	0.430
Waldeyer’s ring	11/114 (9.6%)	3/11 (27.3%)	8/11 (72.7%)	
Gastrointestinal	13/114 (11.4%)	5/13 (38.5%)	8/13(61.5%)	
Other extranodal	32/114 (28.1%)	14/32 (43.8%)	18/32 (56.3%)	
High sIL2R	79/99 (79.8%)	27/79 (34.2%)	52/79 (65.8%)	0.052
High LDH	66/104 (62.9%)	28/66 (42.4%)	38/66 (57.6%)	<0.001
ECOG PS ≥ 2	14/85 (16.5%)	10/14 (71.4%)	4/14 (28.6%)	<0.001
IPI H+HI	31/91 (34.1%)	14/31 (45.2%)	17/31 (54.8%)	0.029
B symptoms	22/87 (25.3%)	10/22 (45.5%)	12/22 (54.5%)	0.058
Treatment				
RCHOP	71/98 (72.4%)	18/71 (25.4%)	53/71 (74.6%)	0.513
RCHOP-like	22/98 (22.4%)	8/22 (36.4%)	14/22 (63.6%)	
Others	5/98 (5.1%)	2/5 (40%)	3/5 (60%)	
Clinical response	24/92 (26.1%)	19/24 (79.2%)	5/24 (20.8%)	<0.001
Death event	54/114 (47.4%)	38/54 (70.4%)	16/54 (29.6%)	<0.001
Pathological features				
Non-GCB (Hans)	77/112 (68.8%)	35/77 (45.5%)	42/77 (54.5%)	<0.001
EBER+	28/112 (25.0%)	15/28 (53.6%)	13/28 (46.4%)	0.011
*MYC* rearrangement+	9/98 (9.2%)	2/9 (22.2%)	7/9 (77.8%)	1.000
*BCL2* rearrangement+	6/97 (6.2%)	1/6 (16.7%)	5/6 (83.3%)	0.665
Double-hit DLBCL+	3/95 (3.2%)	1/3 (33.3%)	2/3 (66.7%)	1.000
High CD163+TAMs	79/113 (69.9%)	33/79 (41.8%)	46/79 (58.2%)	0.005
CD5+	13/113 (11.5%)	4/13 (30.8%)	9/13 (69.2%)	1.000

LDH, lactate dehydrogenase; ECOG PS, Eastern Cooperative Oncology Group performance status (ECOG); IPI, International Prognostic Index for Diffuse Large B-cell Lymphoma (DLBCL); H, high; HI, high–intermediate; Non-GCB, non-germinal center B-cell like by the Hans algorithm; EBER, Epstein–Barr virus-encoded small RNAs (Epstein–Barr Virus (EBV)-positive DLBCL); TAM, tumor-associated macrophages.

**Table 4:** Correlation between the entropy and survival groups.

Overall Survival Group	Entropy < 7.2	Entropy > 7.2	Total
DLBCL Dead within the first 2 years	23/37 (62.2%)	14/37 (37.8%)	37 (100%)
DLBCL Others	57/64 (89.1%)	7/64 (10.9%)	64 (100%)
Total	80/101 (79.2%)	21/101 (20.8%)	101 (100%)

Fisher’s exact test *p* = 0.002.

**Table 5:** Correlation between entropy and clinicopathological characteristics.

	Entropy < 7.2	Entropy > 7.2	*p*-Value
Entropy	6.67 ± 0.38	7.33 ± 0.08	<0.001
Clinical features			
Age > 60 years	57/85 (67.1%)	19/24 (79.2%)	0.320
Sex male	44/85 (51.8%)	13/24 (54.2%)	1.000
Location			
Nodal (+spleen)	38/85 (44.7%)	18/24 (75.0%)	0.071
Waldeyer’s ring	9/85 (10.6%)	1/24 (4.2%)	
Gastrointestinal	11/85 (12.9%)	2/24 (8.3%)	
Other extranodal	27/85 (31.8%)	3/24 (12.5%)	
High sIL2R	60/75 (80%)	15/20 (75%)	0.758
High LDH	47/78 (60.3%)	15/21 (71.4%)	0.449
ECOG PS ≥ 2	6/65 (9.2%)	5/15 (33.3%)	0.028
IPI H+HI	21/69 (30.4%)	7/17 (41.2%)	0.402
B symptoms	17/65 (26.2%)	5/17 (29.4%)	0.767
Treatment			
RCHOP	55/75 (73.3%)	13/18 (72.2%)	0.447
RCHOP-like	17/75 (22.7%)	3/18 (16.7%)	
Others	3/75 (4.0%)	2/18 (11.1%)	
Clinical response	17/72 (23.6%)	7/15 (46.7%)	0.109
Death event	36/85 (42.4%)	15/24 (62.5%)	0.106
Pathological features			
Non-GCB (Hans)	56/83 (67.5%)	18/24 (75.0%)	0.618
EBER+	19/83 (22.9%)	9/24 (37.5%)	0.189
*MYC* rearrangement+	4/74 (5.4%)	1/19 (5.3%)	1.000
*BCL2* rearrangement+	3/73 (4.1%)	0/19 (0%)	1.000
Double-hit DLBCL+	0/71 (0%)	0/19 (0%)	N/A
CD163+TAMs	36.3% ± 25.7	46.9% ± 25.7	0.082
PD-L1+cells	11.8% ± 15.4	14.6% ± 18.4	0.832
IL-10	10.2% ± 12.7	7.4% ± 9.3	0.519
CD5+	10/84 (11.9%)	3/24 (12.5%)	1.000

LDH, lactate dehydrogenase; ECOG PS, Eastern Cooperative Oncology Group (ECOG) performance status; IPI, International Prognostic Index for Diffuse Large B-cell Lymphoma (DLBCL); H, high; HI, high–intermediate; Non-GCB, non-germinal center B-cell like by the Hans algorithm; EBER, Epstein–Barr virus-encoded small RNAs (Epstein–Barr Virus (EBV)-positive DLBCL); TAM, tumor-associated macrophages. N/A, not applicable.

**Table 6:** Correlation between entropy and pathological markers.

	Entropy < 7.2	Entropy > 7.2	*p*-Value
Ki67	15.1% ± 14.9	18.9% ± 13.5	0.264
LMO2	2.7% ± 3.4	2.7% ± 4.4	0.331
MYC	4.9% ± 4.8	5.7% ± 6.9	0.889
MDM2	10.9% ± 8.1	9.5% ± 6.7	0.389
CDK6	4.8% ± 5.8	5.1% ± 9.4	0.133
E2F1	1.9% ± 1.9	1.1% ± 0.8	0.050
BCL2	7.0% ± 9.9	4.4% ± 6.6	0.645
CASP8	7.7% ± 9.3	3.9% ± 3.9	0.156
MYOB	30.5% ± 167.3	2.3% ± 3.1	0.793
TP53	5.8% ± 9.2	3.1% ± 2.0	0.831
cPARP	1.0% ± 1.3	0.6% ± 0.7	0.035
cCASP3	1.4% ± 1.9	0.6% ± 0.5	0.017
ISY1	1.6% ± 2.6	2.4% ± 2.5	0.133
TNFAIP8	39.9% ± 25.1	46.7% ± 28.6	0.496
CSF1R	33.6% ± 27.2	34.0% ± 29.8	0.915
CD163	36.3% ± 25.7	46.9% ± 25.7	0.082
PD-L1	11.8% ± 15.4	14.6% ± 18.4	0.832
IL-10	10.2% ± 12.7	7.4% ± 9.3	0.519

**Table 7:** Overall survival Cox regression multivariate analysis.

Variable	*p*-Value	Hazard Risk	95% CI for Hazard Risk
Entropy	0.005	3.03	1.41–6.51
International prognostic index (IPI)	0.061	0.52	0.27–1.03
Epstein–Barr virus (EBER)	0.012	2.64	1.24–5.66
Cell of origin (Hans)	0.006	3.33	1.42–7.81

Method = entry.

## Data Availability

All data are available upon request to Joaquim Carreras (joaquim.carreras@tokai.ac.jp) and are also shown in the Supplementary File.

## Supplementary Materials

The following supporting information can be downloaded at: https://www.mdpi.com/article/10.3390/cancers18142279/s1.
